# A brain CT-based approach for predicting and analyzing stroke-associated pneumonia from intracerebral hemorrhage

**DOI:** 10.3389/fneur.2023.1139048

**Published:** 2023-06-02

**Authors:** Guangtong Yang, Min Xu, Wei Chen, Xu Qiao, Hongfeng Shi, Yongmei Hu

**Affiliations:** ^1^School of Control Science and Engineering, Shandong University, Jinan, China; ^2^Neurointensive Care Unit, Shengli Oilfield Central Hospital, Dongying, China; ^3^Department of Radiology, Shandong First Medical University and Shandong Academy of Medical Sciences, Jinan, China

**Keywords:** image registration, intracerebral hemorrhage, stroke-associated pneumonia, machine learning, statistical analysis

## Abstract

**Introduction:**

Stroke-associated pneumonia (SAP) is a common complication of stroke that can increase the mortality rate of patients and the burden on their families. In contrast to prior clinical scoring models that rely on baseline data, we propose constructing models based on brain CT scans due to their accessibility and clinical universality.

**Methods:**

Our study aims to explore the mechanism behind the distribution and lesion areas of intracerebral hemorrhage (ICH) in relation to pneumonia, we utilized an MRI atlas that could present brain structures and a registration method in our program to extract features that may represent this relationship. We developed three machine learning models to predict the occurrence of SAP using these features. Ten-fold cross-validation was applied to evaluate the performance of models. Additionally, we constructed a probability map through statistical analysis that could display which brain regions are more frequently impacted by hematoma in patients with SAP based on four types of pneumonia.

**Results:**

Our study included a cohort of 244 patients, and we extracted 35 features that captured the invasion of ICH to different brain regions for model development. We evaluated the performance of three machine learning models, namely, logistic regression, support vector machine, and random forest, in predicting SAP, and the AUCs for these models ranged from 0.77 to 0.82. The probability map revealed that the distribution of ICH varied between the left and right brain hemispheres in patients with moderate and severe SAP, and we identified several brain structures, including the left-choroid-plexus, right-choroid-plexus, right-hippocampus, and left-hippocampus, that were more closely related to SAP based on feature selection. Additionally, we observed that some statistical indicators of ICH volume, such as mean and maximum values, were proportional to the severity of SAP.

**Discussion:**

Our findings suggest that our method is effective in classifying the development of pneumonia based on brain CT scans. Furthermore, we identified distinct characteristics, such as volume and distribution, of ICH in four different types of SAP.

## 1. Introduction

Stroke-associated Pneumonia (SAP) is a serious complication for patients with intracerebral hemorrhage (ICH), leading to increased hospitalization time, medical expenses, and mortality rates (1–4). The causes of SAP can be categorized as central or non-central factors, with the former including disturbance of consciousness and bulbar palsy, and the latter including bed rest, pulmonary edema, and pre-existing chronic respiratory conditions such as COPD, bronchiectasis, and pulmonary fibrosis (5, 6). While tracheal intubation can protect the airway, it also increases the risk of ventilator-associated pneumonia (VAP) (7, 8). To effectively identify high-risk groups of pneumonia in patients with acute and severe ICH, clinicians commonly use pneumonia CT scans to review lung infections (9). Accurately identifying pneumonia-prone patients is essential to guide clinical decisions regarding tracheal intubation and to provide timely interventions to reduce the risk of pneumonia in this vulnerable population, especially for inexperienced healthcare providers.

Risk factors associated with pneumonia include immunosuppression, dysphagia, age, sex, smoking, stroke severity, stroke type, hypertension, diabetes, history of chronic respiratory disease, and history of atrial fibrillation (10, 11), which are usually referred as baseline (clinical) data. Previous studies suggest that predicting the risk of a lung infection after stroke can help doctors select interventions to reduce morbidity in high-risk patients (4). Ji et al. (12) developed an SAP risk model “ICH-APS” based on the patients' baseline data, which could effectively predict pneumonia after ICH, especially for patients whose hospitalization time was more than 48 h. Yan et al. (13) used the permutation method to select the characteristics and finally constructed a logical regression model called “ICH-LR2S2” using nine patient characteristics, including dysphagia, age, sex, and fasting blood glucose. But baseline data is hard to collect and could not build a relationship with ICH distribution. If the ICH region corresponds to the relevant brain area, it will have a meaningful and positive effect on the study of the generation mechanism and progression of SAP (14). CT is the most common experimental method for ICH patients, and it is feasible to locate bleeding areas and distinguish between left and right hemispheres using CT images due to the different Hounsfield unit (Hu) values; however, CT images cannot accurately label the brain structure; therefore, high-quality brain MRI images are required. Brain MRI is being increasingly used in research and clinical medicine to obtain high-quality images of the brain's anatomical structure, providing detailed information for clinical diagnosis and biomedical research (15, 16). Medical image registration has important clinical application value: the registration of medical images obtained by various or the same imaging methods is used for medical diagnosis as well as in formulating surgical plans, formulating radiation therapy plans, tracking pathological changes, and evaluating treatment effects (17–20). However, thus far, no research on the relationship between ICH and pneumonia through medical image registration technology has been reported.

**Figure 1:**
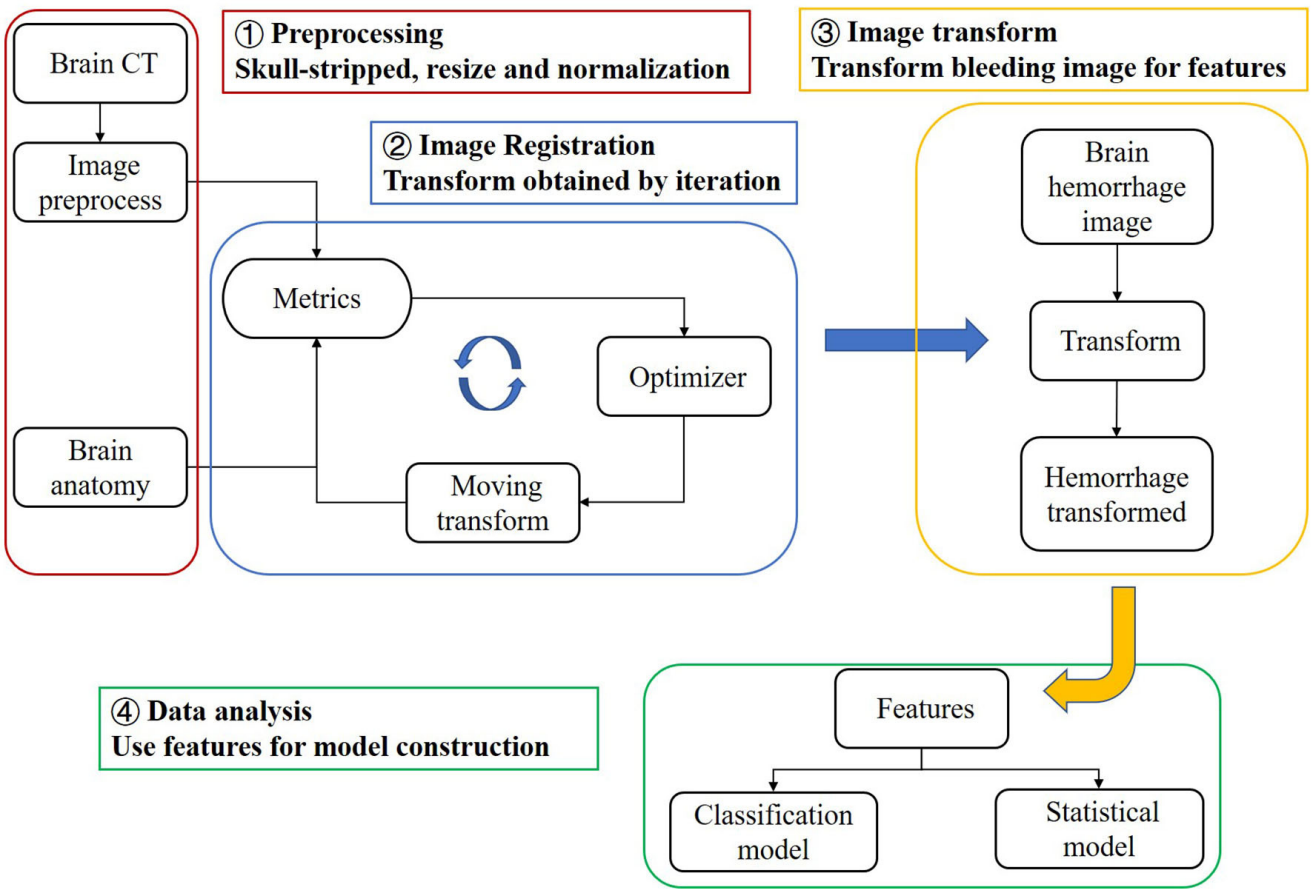
Research workflow of this study.

In this study, we propose a registration method to match brain CT images with MRI images representing the brain's anatomical structure. This allows us to obtain the anatomical distribution characteristics of the hemorrhage area, which we refer to as the “bleeding distribution feature”. Since the hemorrhagic mass can squeeze the patient's brain tissue, we also determine the “bleeding squeezing feature” based on the relationship between the total bleeding magnitude and the patient's brain tissue. Using these features, we establish three machine learning models to predict the severity of SAP using brain CT instead of lung CT in clinical situations. We can further discuss important features by implementing feature selection. Additionally, we superimpose transformed binary bleeding area images from four pneumonia categories to build a statistical model and probability atlas. This visualization and analysis of the distribution of cerebral hemorrhages in different pneumonia categories can be beneficial for diagnosis and treatment. The flow chart of this study is shown in Figure 1.

## 2. Materials and methods

### 2.1. Datasets

Our dataset comprises 244 brain CT images, each with a corresponding segmented image of ICH. Experienced neurologists annotated the ICH area using ITK-Snap software, while the patient's name, gender, underlying disease, admission time, corresponding treatment, and other basic information were recorded in detail. Following a previous study (21), we assessed the extent of pneumonia in patients with early ICH (1–4 days of onset) by evaluating the lung involvement area on chest CT (classified as mild, moderate, or severe based on involvement percentages of 1–25, 26–50, and 51–100%, respectively). All chest CT images were independently reviewed by two radiologists with more than 10 years of experience, who were blinded to clinical and laboratory findings. The images were subsequently categorized into 19 cases of severe pneumonia, 47 cases of moderate pneumonia, 77 cases of mild pneumonia, and 101 cases of no pneumonia.

After obtaining informed consent from the local ethics committee (Q/ZXYY–ZY–YWB–LL202243), the CT images were used for further research.

### 2.2. MRI atlas

**Figure 2:**
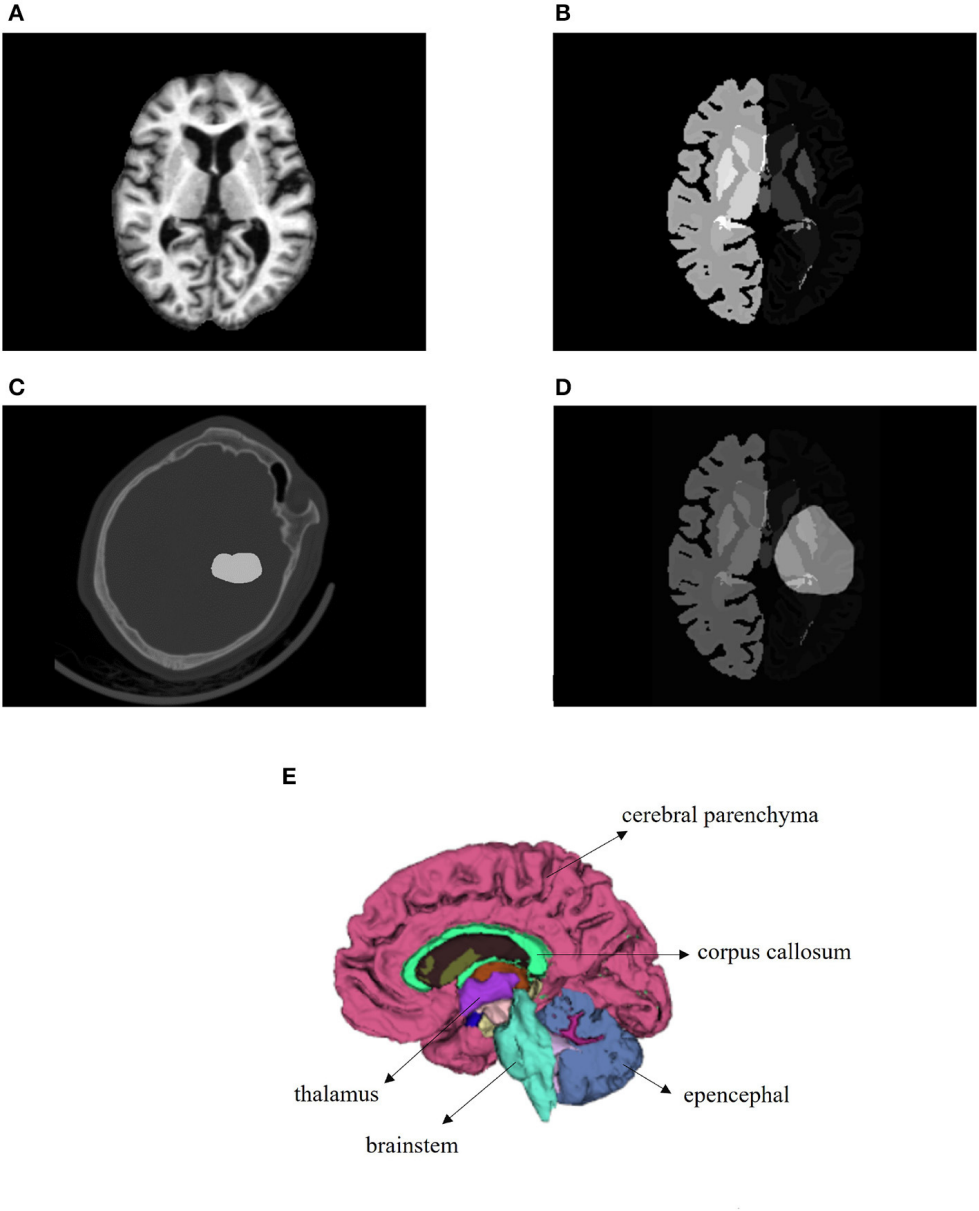
Images utilized in this paper. **(A)** Brain MRI used in the registration as a reference image. **(B)** Brain anatomical structure MRI having 35 regions. **(C)** One brain CT sample with ICH area labeled. **(D)** Transformed bleeding image shown with brain anatomical image. **(E)** Three-dimensional schematic diagram of the anatomical structure of the brain.

In this study, a normal human brain MRI image was chosen as the reference image for registration, and its corresponding anatomical structure image was used as a template for analysis. The MRI image used in this study was obtained from the OASIS project, which contains 35 anatomical structure divisions. Any area in the image with a grayscale value of 0 was considered a blank area (22). To process the MRI image, Hoopes et al. (23) utilized FreeSurfer software to remove the skull and align the images. The image was then divided into 35 brain anatomical regions, each assigned a grayscale value ranging from 1 to 35. Table 1 provides a list of the specific structure names and their corresponding gray values. Additionally, Figures 2A, B illustrates the brain MRI image and the corresponding brain anatomical structure MRI. Figure 2E presents the 3D diagram of the anatomical structures resulting from the MRI images.

**Table 1:** Brain anatomical structure reference.

**Gray value**	**Brain anatomy**	**Gray value**	**Brain anatomy**	**Gray value**	**Brain anatomy**	**Gray value**	**Brain anatomy**
1	Left-Cerebral-White-Matter	10	Left-Pallidum	19	Left-Choroid-Plexus	28	Right-Putamen
2	Left-Cerebral-Cortex	11	3rd-Ventricle	20	Right-Cerebral-White-Matter	29	Right-Pallidum
3	Left-Lateral-Ventricle	12	4th-Ventricle	21	Right-Cerebral-Cortex	30	Right-Hippocampus
4	Left-Inf-Lat-Ventricle	13	Brain-Stem	22	Right-Lateral-Ventricle	31	Right-Amygdala
5	Left-Cerebellum-White-Matter	14	Left-Hippocampus	23	Right-Inf-Lat-Ventricle	32	Right-Accumbens
6	Left-Cerebellum-Cortex	15	Left-Amygdala	24	Right-Cerebellum-White-Matter	33	Right-Ventral-DC
7	Left-Thalamus	16	Left-Accumbens	25	Right-Cerebellum-Cortex	34	Right-Vessel
8	Left-Caudate	17	Left-Ventral-DC	26	Right-Thalamus	35	Right-Choroid-Plexus
9	Left-Putamen	18	Left-Vessel	27	Right-Caudate		

### 2.3. Acquisition of features

In this study, we used the bleeding distribution and extrusion features to construct our models. In order to obtain the bleeding distribution feature, we matched preprocessed brain CT to the brain MRI image to get the deformation field of the process and applied the deformation field to the corresponding ICH segmented image (binary bleeding image) of the patient to generate the transformed image. The transformed bleeding image was used to extract the bleeding distribution feature.

#### 2.3.1. Image preprocessing

**Figure 3:**
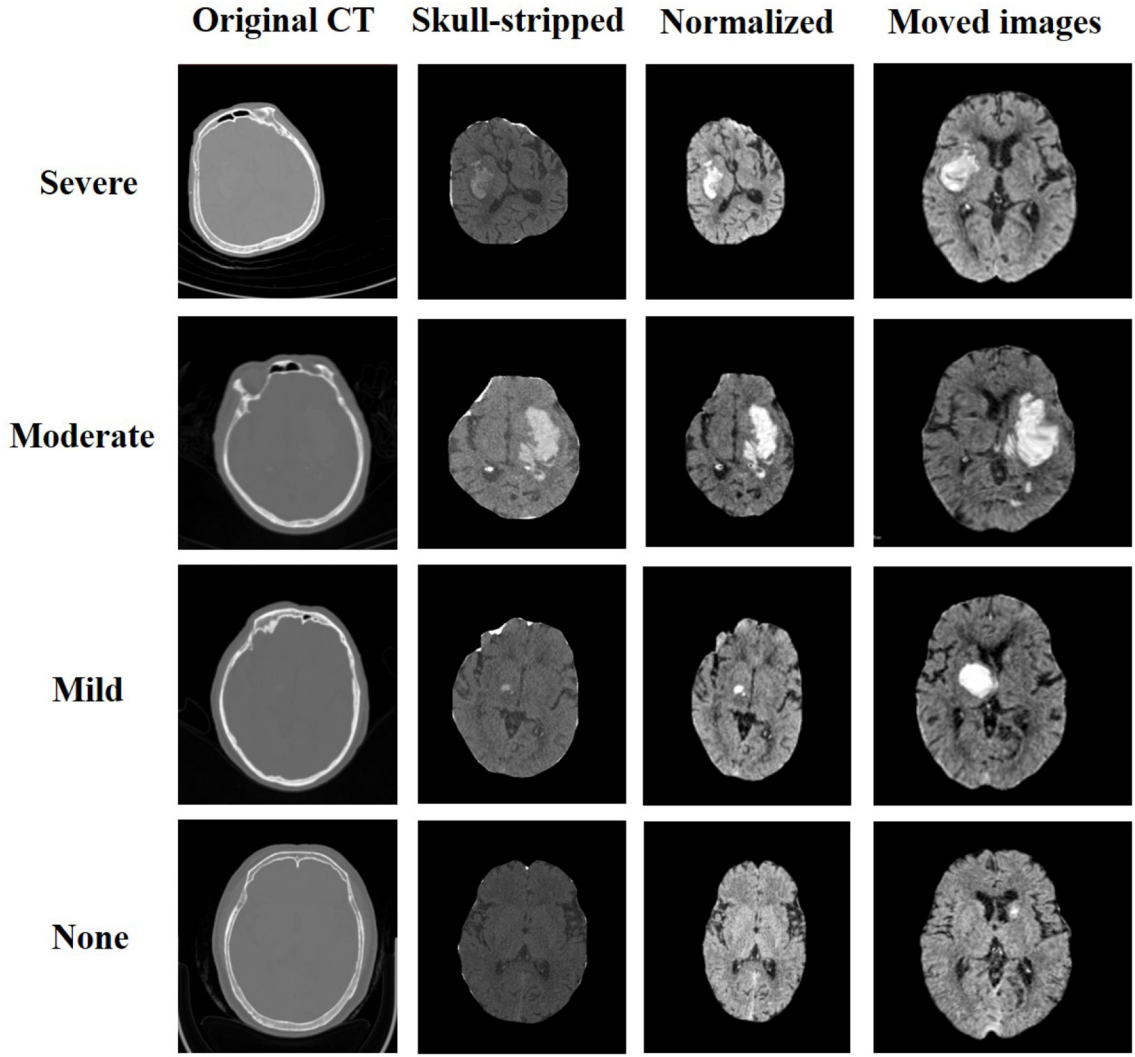
Resulted images of preprocess and registration.

To improve the image registration results, several pre-processing steps, such as skull-stripping, image normalization, and resampling, were performed on the original brain CT image (24). Since the reference MRI image used as a fixed image only shows brain tissue, it was necessary to remove the skull from the patient's original CT image. To achieve this, we utilized the SegmentEditorExtraEffects and SurfaceWrapSolidify extension modules in the 3D Slicer software (25). Specifically, we created a new segment in the segmentation editor from the skull segmentation, determined the threshold to initially segment the skull, used the islands method to remove small spots caused by image noise, and applied the Wrap Solidify effect to segment the inner area of the skull as a mask. Finally, we used the mask as our output after removing the skull from the CT image. To process a large number of samples efficiently, we developed a Python script for the 3D Slicer to automate the skull-stripping operations for all samples, thereby reducing time and labor. Moreover, we used min-max normalization to preprocess the CT image after skull-stripping. The normalization equation used is as follows:


(1) $$\begin{array}{l} v=\left(v-min\right)/\left(max-min\right) \end{array}$$

where *v* is the voxel value of the image and min and max are the minimum and maximum voxel values of the image, respectively. Since the obtained brain CT image and reference MRI image had different dimensions, we resampled the image to match the dimensions of the reference MRI image. The resulting images are depicted in Figure 3.

#### 2.3.2. Image registration

According to the characteristics of our data, we performed image transformation by applying both rigid and non-rigid registration methods (26). Rigid transform is a type of transformation that preserves the shape of an image by including translation, rotation, and scaling. It can be used to align images rigidly. Non-rigid transform refers to a type of transformation that can be used to deform, warp, or morph images. It can deform different regions of the image but cannot preserve the overall shape of the image. During the registration process, the CT image was considered as the moving image, denoted as *I*_*m*_(*v*), while the MRI image was regarded as the fixed image, denoted as *I*_*f*_(*v*), where *v* represents the voxel of the image. The moving image was iteratively transformed to find the most suitable transform *T*(*v*) that matches the fixed image. The objective was to minimize the mutual information between the two images, which can be expressed as follows:


(2) $$\begin{array}{l} min_{x=3}MI\left(I_{m}\left(v\right),I_{f}\left(v\right)\right) \end{array}$$

Here, the parameter *x* = 3 indicates that our image is three-dimensional. Since the moving and fixed images had different origins and orientations, we applied a rigid transformation to translate and rotate the original image without changing its size or internal structure. Next, we used non-rigid transformation to precisely match the CT image to the reference brain MRI image, using the “bspline” method and inputting the result into the previous image filter. In this process, we adopted a multi-resolution strategy to construct a resolution pyramid, and each resolution layer performed a maximum number of iterations to obtain the optimal result.


(3) $$\begin{array}{l} T=T_{rigid}+T_{nonrigid} \end{array}$$

In our study, we utilized mutual information (MI) as the evaluation metric for each iteration of our registration process (27). MI is a versatile metric that calculates the mutual information between two images, based on the correlation of the probability density distribution (PDF) of the intensity from the fixed and moving images. MI measures the amount of information that a random variable (such as image intensity in one image) tells another random variable (such as image brightness in another image), without requiring knowledge of the actual form of the correlation. Therefore, it is particularly suitable for multimodal image pairs and single-mode images. The output image was determined as the moving image with the highest MI score (28, 29). The images before and after the registration of one patient are presented in Figures 2C, D.


(4) $$\begin{array}{l} MI\left(X,Y\right)=I_{binned}\left(X,Y\right)=\underset{ij}{\sum }p\left(i,j\right)log\left(\frac{p\left(i,j\right)}{p_{x}\left(i\right)P_{y}\left(j\right)}\right) \end{array}$$

Where $p_{x}(i)=\int_{i}dx\mu_{x}(X)$, $p_{y}(i)=\int_{i}dx\mu_{x}(X)$, and $p(i,j)=\int_{i}\int_{j}dxdy\mu (x,y)$ and $\int_{i}$ means the integral over bin *I* and μ means the marginal densities.

#### 2.3.3. Bleeding area transformation

After completing the registration of the patients' CT images, we obtained the deformation field. The image of the bleeding area denoted by the doctor was binarized, where 1 and 0 represented the bleeding and non-bleeding areas, respectively. We performed the same preprocessing operations as for the CT images except for skull-stripping on the original bleeding area image, to make the transformed bleeding image consistent with the reference brain anatomy MRI. The transformed bleeding image was then overlayed on the MRI image, as shown in Figure 2D.

#### 2.3.4. Obtaining bleeding distribution features

After transforming the bleeding area image, we obtained a binary image in which the value 1 represents bleeding and 0 represents non-bleeding. We then identified the position of each voxel with a value of 1 in the transformed image and determined the corresponding voxel value (ranging from 1 to 35) in the reference MRI image. Next, we identified the brain structures covered by the voxel points representing the hemorrhage area. By processing all voxel points, we obtained the number of voxels representing bleeding on a specific anatomical structure *BNum*_*i*_. We then calculated the proportion of bleeding in all 35 anatomical structures partitioned for the patient, represented by *BNum*_*i*_, relative to the total number of voxels in that structure, *AllNum*_*i*_, using the following equation:


(5) $$\begin{array}{l} Bd_{i}=BNum_{i}/AllNum_{i} \end{array}$$

This allowed us to quantitatively analyze the distribution of bleeding in different brain structures. The results of this analysis are presented in our study.

#### 2.3.5. Obtaining bleeding extrusion features

We quantified the cerebrospinal fluid and brain parenchyma areas in the original CT image based on the Hu value during CT imaging of different tissues and represented them using the number of voxels. We also calculated the total volume of the bleeding area. Additionally, we constructed five specific proportional characteristics: cerebrospinal fluid to the brain parenchyma, cerebrospinal fluid to the brain parenchyma and cerebrospinal fluid, hemorrhage to cerebrospinal fluid, hemorrhage to the brain parenchyma, and hemorrhage to cerebrospinal fluid and the brain parenchyma. In total, we obtained eight bleeding volume and extrusion features that characterize the extent to which the hemorrhagic mass affects the surrounding brain tissue. These features are referred to as bleeding extrusion features.

We stored all the obtained features, the ICH distribution, and hemorrhage extrusion features in the file. The summary and meaning of all features can be roughly distributed into three classes, namely, L', which indicates the hemorrhagic volume, L/All' presented as “/”, indicating the proportion of the bleeding volume to brain tissue, and numbers 1–35 indicating the proportion of the bleeding volume in the brain anatomical structure. Specific meanings are given in Supplementary Table 1.

### 2.4. Construction of classification and statistical models

The obtained features were used to construct machine learning models for classification prediction and feature selection. A probability map was then generated to analyze the distribution characteristics of bleeding areas for four types of pneumonia.

#### 2.4.1. Classification model

To analyze the data, we combined the bleeding distribution feature and bleeding extrusion feature and trained three classical machine learning models, namely logistic regression, support vector machine (SVM), and random forest, to classify and predict whether patients have pneumonia symptoms. The labels referring to the degree of pneumonia progression were severe, moderate, mild, and no symptoms of pneumonia. We used several indicators to evaluate the model, including area under the curve (AUC), accuracy, sensitivity, and specificity. Sensitivity is defined as the ratio of true positives to all positive samples, and specificity is defined as the ratio of true negatives to all negative samples. For each model, we evaluated the average metric of 10-fold cross-validation.

The classification problem of with or without pneumonia (SAP) can be viewed as a typical binary classification problem based on the cerebral hemorrhage situation. The feature combinations could reflect the contribution of different characteristics to the classification problem. Before entering the model, data standardization was required to improve the classification effect of the model.

Furthermore, since moderate-to-severe pneumonia can lead to prolonged hospitalization and increase the risk of poor patient outcomes (30), we divided the patients into two categories: SAP above moderate level and the others and performed a two-category prediction problem. We used three different machine learning models, and the features were treated the same as the above classification task. Data standardization was performed, and the mean value of the metrics of the 10-fold cross-validation was used to instruct the classification problem.

#### 2.4.2. Statistical model

In addition to the classification task, we developed a statistical model based on the pneumonia classification to present and analyze the data in the form of a probability map. The specific method for constructing the probability map for one category involved superimposing the transformed hemorrhagic area images of all patients with the same SAP type. During the registration process when matching the brain CT and brain MRI, a transformation was generated for each patient. Multiple binarized images were then superimposed to create the probability map, with a gray value of 0 in some places and the largest voxel not exceeding the number of people with the particular pneumonia type. We compared the gray value of all voxels in the final image to the number of people in the category to obtain the probability map. To visualize the distribution characteristics of the hemorrhagic areas of various types of pneumonia more intuitively, we used the 3D Slicer software to superimpose the obtained probability maps with the brain MRI image. The original hemorrhage area images were not directly added to obtain the probability map because they could not be correlated with the brain anatomy MRI.

Furthermore, we analyzed the bleeding volume of each type of patient and constructed a box plot to examine the relationship between the development of pneumonia and the bleeding volume.

## 3. Results

We extracted a total of 35 bleeding distribution features, which included bleeding extrusion features, as well as the volumes of the cerebrospinal fluid, brain parenchyma, and hemorrhage mass (represented by the number of voxels). The remaining five features were represented on a scale of 0 to 1. To ensure that each feature contributed equally to the analysis, we performed standardization operations on the features based on their eigenvalues. Figure 3 displays the samples that were selected from each category of pneumonia. For each sample, both the preprocessed image and the final image after registration are shown.

### 3.1. Classification model

The evaluation metrics are AUC value, accuracy, sensitivity, and specificity. For predicting SAP, the logistic regression model achieved an AUC of 0.79, with all four indexes above 0.73, and the specificity index being the highest among the three classifiers. The SVM model achieved the highest accuracy and sensitivity of 0.76 and 0.77, respectively. The random forest model performed the best in terms of AUC, which was above 0.8, and all other indexes were greater than 0.7. It should be noted that the ICH-APS-A model (12) achieved an AUC of 0.76, while the ICH-LR2S2 model (13) obtained an AUC of 0.78 in their respective test cohorts. This suggests that our method has performed well. Table 2 presents the performance evaluation results of the three classification models for predicting the occurrence of SAP.

**Table 2:** Metrics of machine learning models for predicting SAP.

**Model**	**AUC**	**Accuracy**	**Sensitivity**	**Specificity**
Logistic regression	0.79	0.75	0.75	0.74
SVM	0.79	0.76	0.77	0.72
Random forest	0.82	0.73	0.76	0.70

**Figure 4:**
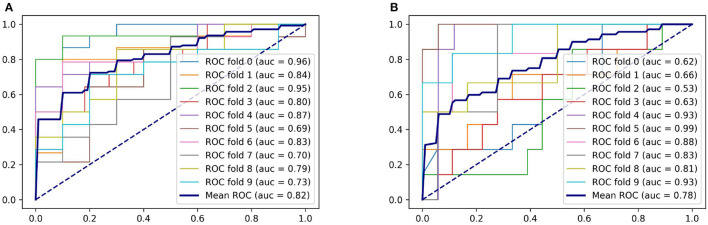
ROC curves of the random forest model. **(A)** For predicting SAP. **(B)** For predicting SAP above the moderate level.

For predicting SAP above a moderate level, the logistic regression model achieved an AUC of 0.77, with accuracy and specificity above 0.7. The SVM model achieved the best performance on accuracy and sensitivity of 0.75 and 0.74, respectively. The random forest model had an AUC and specificity of 0.78, the highest among the three models. Figure 4 displays the ROC curve of the random forest model, which demonstrated the highest performance. The ROC curves of the other two models are available in Supplementary material. Table 3 shows the experimental results of the two-classification problems for differing SAP above the moderate level.

**Table 3:** Metrics of machine learning models for predicting SAP above the moderate level.

**Model**	**AUC**	**Accuracy**	**Sensitivity**	**Specificity**
Logistic regression	0.77	0.72	0.67	0.74
SVM	0.77	0.75	0.74	0.74
Random forest	0.78	0.75	0.66	0.78

The obtained AUCs were all above 0.75 for both classification problems, demonstrating that the severity of pneumonia can be predicted by the features extracted from our method.

### 3.2. Statistical model

**Figure 5:**
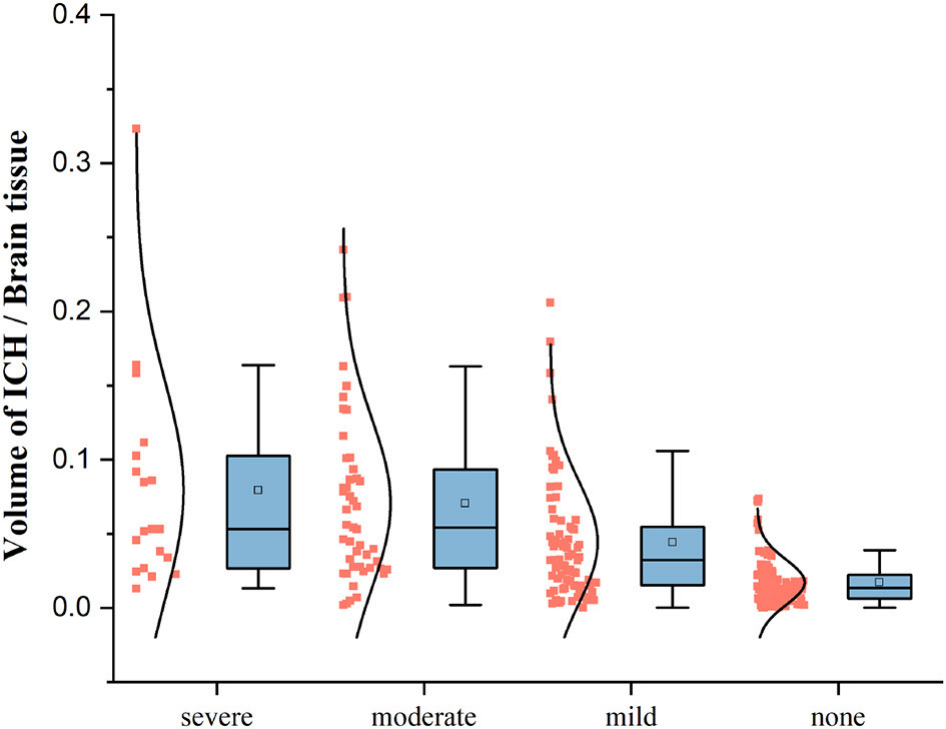
The box plot of the ratio of bleeding volume to total volume based on four SAP types.

The bleeding volume in patients with pneumonia symptoms is generally distributed below 20% of the brain tissue volume, with a higher density of patients having a bleeding volume ratio of less than 10%. However, patients without pneumonia symptoms also show bleeding volume ratios below 10% of the brain tissue volume. Thus, additional features are required to effectively classify pneumonia symptoms. The box plot in Figure 5 indicates that the bleeding volume alone is not a reliable indicator for classifying pneumonia symptoms.

**Figure 6:**
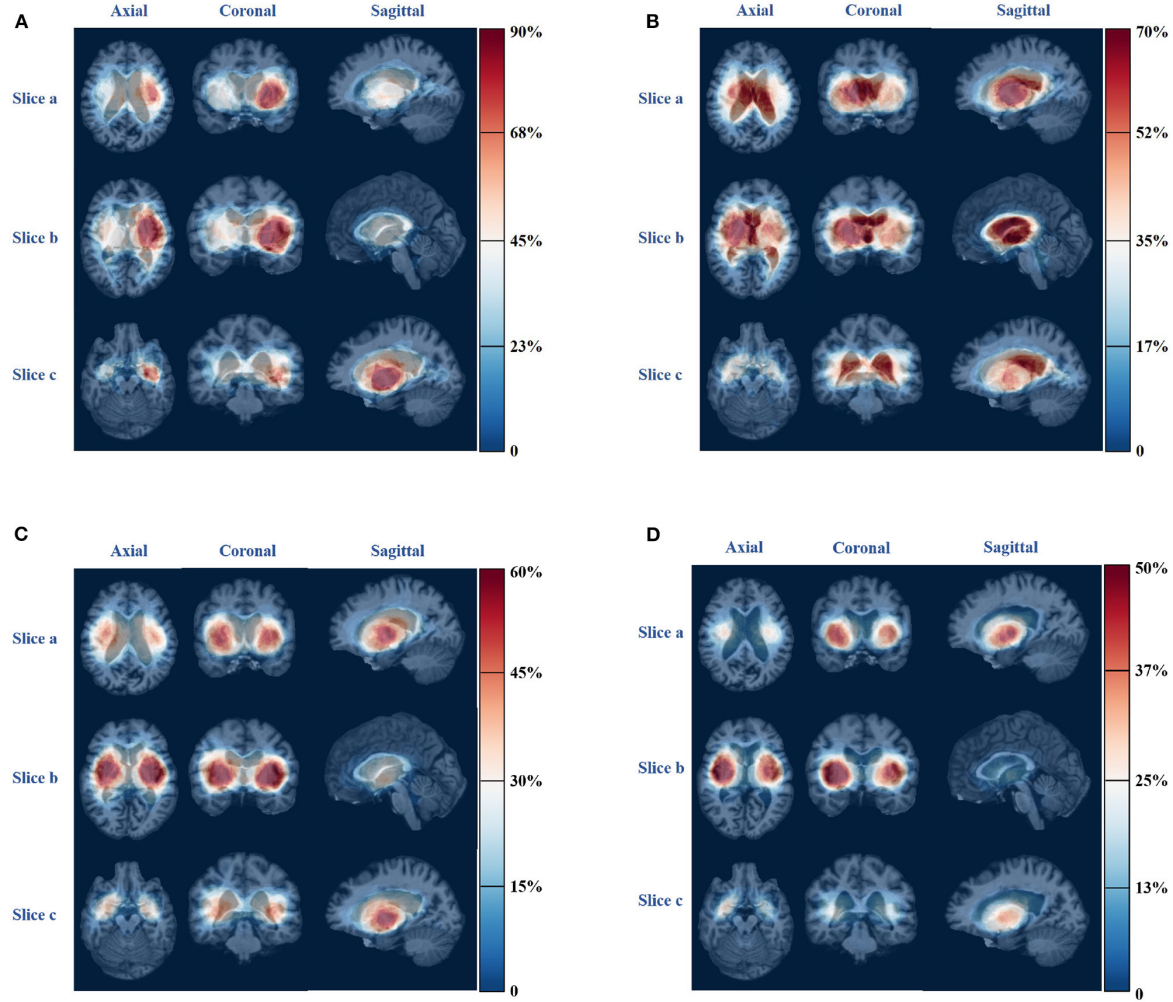
Bleeding probability map of four types of pneumonia. **(A)** Severe pneumonia. **(B)** Moderate pneumonia. **(C)** Mild pneumonia. **(D)** No pneumonia.

For patients with severe pneumonia, there is an unbalanced distribution of hemorrhagic areas in the left and right cerebral hemispheres. The probability of hemorrhage in some areas of the left brain is high, with a probability above 0.6, while the probability of hemorrhage in some areas of the right brain is low, with a probability of approximately 0.3. In contrast, for patients with mild pneumonia or no pneumonia symptoms, there is no significant imbalance in the distribution of hemorrhagic areas between the left and right brain. The probability map can be a useful tool for analyzing the distribution characteristics of hemorrhagic areas in different types of pneumonia and can aid in diagnosing and treating patients with pneumonia. Figure 6 shows the probability map for the four pneumonia categories, which provides valuable insights into the distribution of hemorrhagic areas in different brain regions.

## 4. Discussion

We constructed classification and statistical models based on the obtained features and combined them with the clinical expertise of physicians to correlate the development of SAP in patients and the distribution of cerebral hemorrhages.

### 4.1. Novelty of our method

For predicting pneumonia infection after ICH, previous studies mostly focused on the patients' baseline data and aimed to build a risk model. Their risk model used AUC as an evaluation index and achieved good performance, ICH-LR2S2 (13) was constructed based on nine patient features and used an external validation cohort to evaluate the model. The overall performance of ICH-LR2S2 was AUC = 0.784. The ICH-APS (12) model achieved an AUC of 0.76 on its validation cohort and was also built by baseline data. Our logistic regression model was established on the features extracted by the registration method from the MRI atlas and achieved a good performance of AUC = 0.79 on the validation set for predicting SAP. There is a fact that the patients' baseline data is hard and time-consuming to collect and preprocess, such as data filling and cleaning, for our proposed method, the only input was the segmented ROI of brain CT which could rapidly offer risk score and the classified results after CT examination if an accuracy segmentation algorithm is matched with our model. It is worth mentioning that, ICH-LR2S2 and ICH-APS had a lot of data in the training phase, our database still needs to be expanded and methods should be improved and practiced in clinical situations.

### 4.2. Feature selection

**Figure 7:**
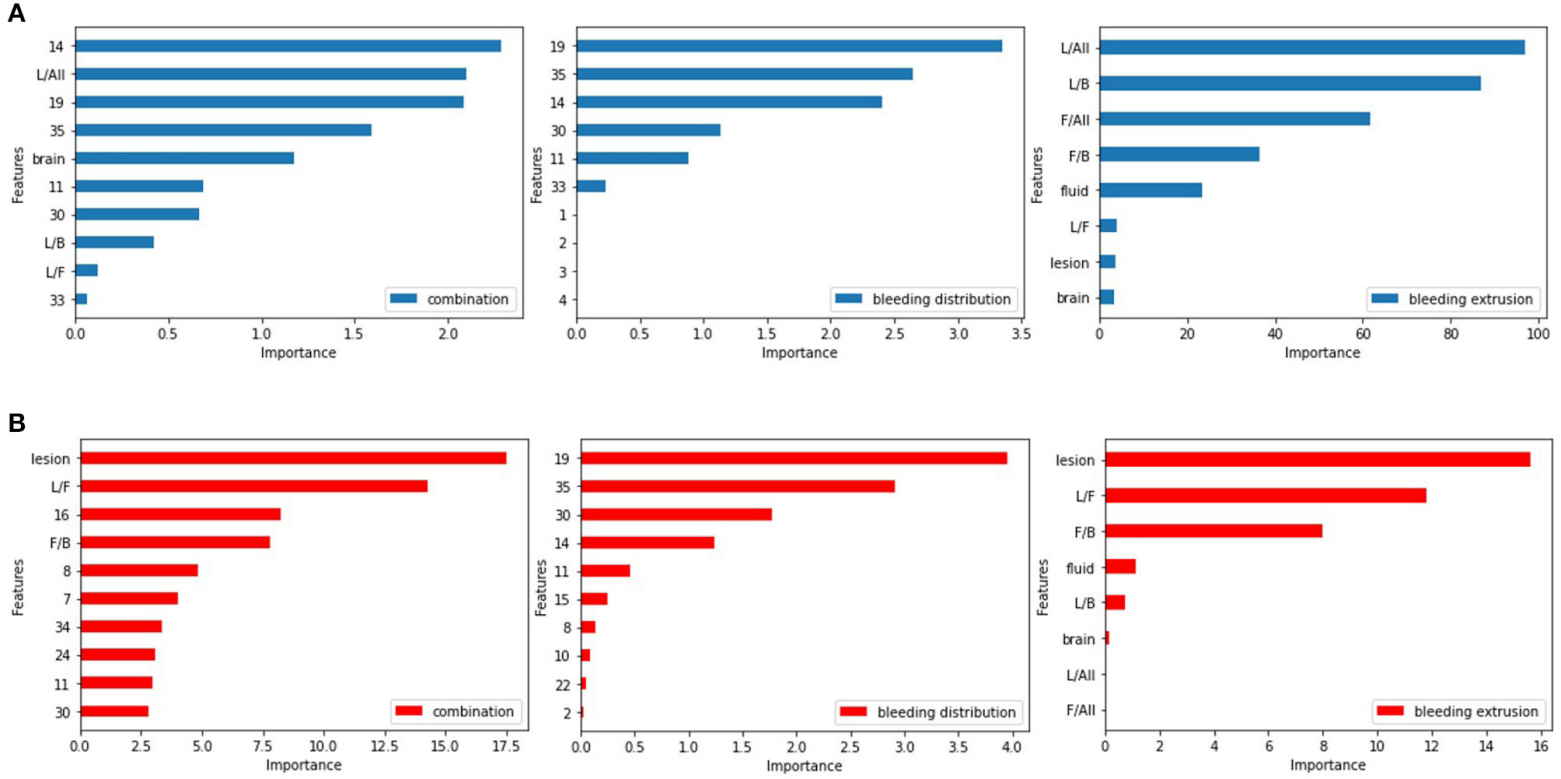
Feature weights of the logistic regression model. **(A)** For predicting SAP. **(B)** For predicting SAP above the moderate level.

Previous studies have shown that using L1 regularization to penalize the logistic regression model can significantly impact model performance by achieving critical factors (31, 32). In our study, we inputted two types of features and their combinations into the model and implemented 10-fold cross-validation. By considering the feature weights from the regression coefficients shown in Figure 7, we were able to determine the factors and brain regions that occupied important weights in our logistic model.

Among the bleeding distribution features, the left-hippocampus, left-choroid-plexus, right-choroid-plexus, third-ventricle, and right-hippocampus had larger weights than others, indicating that ICH in these regions is associated with a greater risk of pneumonia infection. The involvement of these areas can cause swallowing dysfunction or disturbance of consciousness in the patient (33). Among the bleeding extrusion feature, we found that hemorrhage and cerebrospinal fluid volume can contribute to the development of pneumonia. The weight of the cerebrospinal fluid is relatively large due to its liquid nature, and there is circulation and absorption of the cerebrospinal fluid (34). Figure 7A shows the feature weights for predicting SAP.

For predicting SAP above moderate level, the brain regions that contributed more to the model were the left-choroid-plexus, right-choroid-plexus, right-hippocampus, left-hippocampus, and left-accumbens, usually located in the basal ganglia region. When involved, swallowing or conscious function will be affected (35), and the right cerebellar white matter involvement affects ataxia function (36). For the bleeding extrusion feature, the hemorrhage volume and cerebrospinal fluid and its proportion with the brain tissues play a significant role in the classification problem. The mass effect occurs during ICH due to the cerebrospinal fluid's nature and its circulation and absorption functions. The cerebrospinal fluid plays a major buffer function (37), and the image shows the shrinkage of the ventricular system, whereas the amount of bleeding and the mutual ratio of the cerebrospinal fluid and brain tissue change significantly. Figure 7B shows the feature weights for predicting SAP above the moderate level.

Through feature selection, we surprisingly found that some anatomical regions implemented by ICH cause higher risk scores of SAP, including the choroid-plexus, hippocampus, and third-ventricle. If the hemorrhage mass affected the basal ganglia region, it increased the risk of pneumonia developing to more than moderate. Additionally, whether the cerebrospinal fluid was affected by hemorrhage mass was a critical factor in the fact of feature selection.

### 4.3. The distribution of cerebral hemorrhage

The distribution of ICH in severe pneumonia shows a left-right imbalance, as shown in Figure 6A. More than 70% of patients with severe pneumonia had hemorrhage in regions such as left-cerebral-white-matter, left-pallidum, left-putamen, left-thalamus, left-ventral-dc, and left-cerebral-cortex, which are all located in the left half of the brain. Since the left hemisphere is mostly the dominant hemisphere, involvement of this hemisphere results in more severe disease, combined with disturbance of consciousness, swallowing dysfunction, and inability to expectorate sputum, leading to poor airway protection and severe pulmonary infection (38).

For patients with moderate pneumonia, more than 50% had hemorrhagic clots in the right-cerebral-white-matter, right-lateral-ventricle, right-thalamus, right-pallidum, third-ventricle, left-lateral-ventricle, right-caudate, and left-choroid-plexus, which are mostly located in the right half of the brain. The volume of hemorrhage in the cerebrospinal fluid was also found to be greater in patients with this type of pneumonia compared to those with the other three types. The right hemisphere is the non-dominant hemisphere, which has little impact on the patient's consciousness and allows limited airway protection ability.

For patients with mild pneumonia, only a small portion of the probability map exceeds 0.5, indicating that there is no obvious clustering in the distribution of ICH in this category. More than 40% of the patients had bleeding clumps in regions, such as right-cerebral-white-matter, right-cerebral-cortex, left-cerebral-white-matter, left-cerebral-cortex, right-putamen, left-putamen, left-pallidum, right-pallidum, right-thalamus, and left-thalamus, and there was no apparent imbalance in the distribution of the left and right brains.

For patients without symptoms of pneumonia, hemorrhages were found in regions such as the right-cerebral-cortex, right-putamen, right-cerebral-white-matter, and right-pallidum in more than 40% of patients. Hemorrhages occurred in the right white matter and right cerebral cortex in more patients than in the left-brain matter.

### 4.4. Hemorrhage volume and pneumonia classification

According to the data box plot of the ratio of the bleeding volume to brain tissue in each category of patients, we found that the upper limit, upper quarter, mean and maximum value of the data exhibited an increasing trend according to the deepening of the development of SAP. Additionally, the data in the category without pneumonia symptoms are all lower than the three categories with pneumonia symptoms except for the lower limit point. There is a significant difference, indicating that if the patient does not show pneumonia symptoms, the probability of bleeding will not be higher than 10% of the brain tissue. If the ICH mainly occurs in a functional area, especially when the patient's consciousness, swallowing function, or expectoration reflex is affected, pulmonary infection is more likely to occur.

Moderate-to-severe pneumonia with cerebral hemorrhage significantly increases hospitalization and medical expenses and can aggravate brain damage and cause other complications (30). We divided the data into two types: SAP above moderate level and others. There are obvious differences in the box plot data of ICH between the two types. The maximum value, upper limit, upper quartile, mean, median, lower quartile, and lower limit in the data of patients with moderate and severe pneumonia are higher than those of no, mild pneumonia. This indicates that with the development of pneumonia, the volume of bleeding gradually increased. For the binary classification problem, according to the weights of the features and the deduction that patients in each category can have bleeding areas with volumes less than 10% of brain tissue, we cannot make an effective prediction using a single factor of the hemorrhage volume; we need to consider multiple features to predict pneumonia. Protection of the airway is dependent on the level of consciousness and swallowing function. The functional areas affecting the patient's consciousness and swallowing function are mainly distributed in the ascending reticular activation system, bilateral basal ganglia, posterior cranial nerves, and other related areas (35). Therefore, the influence of the structure can better predict the risk of early pulmonary infection in patients by effectively combining the amount of bleeding with the bleeding site, evaluating the effect of mass on each area, and taking relevant measures in a timely manner for a better recovery effect and quality of life.

### 4.5. Technical limitations

The precision of the registration process is critical to the accuracy of our conclusion analysis, as the hemorrhage distribution feature we obtained is based on the image transformation of registration technology. We utilized non-rigid transformation based on continuous optimization iteration, and technical precision can be improved. In recent years, deep learning technology has significantly advanced the development of medical image registration, such as BIRNet. Fan et al. (39) proposed a fully convolutional network subject to dual guidance, including ground truth and image dissimilarity guidance, which demonstrated high registration accuracy and efficiency. For unsupervised learning, Balakrishnan et al. (40), Krebs et al. (41), Vos et al. (42), and Gierlichs et al. (28) proposed end-to-end networks that estimate deformable transformations by maximizing the similarity between image pairs without real deformations. Using deep networks to improve image registration in our study facilitated more accurate results and improved model accuracy.

While our data comes from only one hospital and is limited in size, our method needs to be validated on an external cohort and evaluated for clinical application. Thus, limitations are associated with a single center and species. Collecting data from multiple centers could yield more interesting results, especially considering that most Asians are right-handed.

## 5. Conclusion

To the best of our knowledge, no study has been reported on the prediction and analysis of SAP based on the distribution characteristics of the ICH area. In this study, we utilized an MRI atlas that could clearly represent 35 anatomical brain regions and creatively combined our data with MRI through medical image registration. We constructed machine learning models to detect the occurrence and development of SAP using patient brain CT scans, which to a certain extent, reduced dependence on lung CT and clinical doctors.

Our findings suggest that hemorrhage in specific brain regions, such as the left-choroid-plexus, right-choroid-plexus, right-hippocampus, and left-hippocampus, were more likely to affect the development of pneumonia. We determined the distribution characteristics of the ICH in patients with various types of pneumonia by using a probability map and box plot. Specifically, patients with severe pneumonia had more ICH in the left cerebral hemisphere (dominant side), whereas those with moderate pneumonia had more ICH in the right cerebral hemisphere and cerebrospinal fluid. The volume of the patients' ICH played an important role in the occurrence and development of pneumonia, as shown by significant differences in the amount of bleeding in different categories of patients in the box plot, which mainly displayed maximum, average, and median indicators.

In summary, our study presents a novel method for predicting and analyzing SAP based on the distribution characteristics of the ICH area in brain CT scans. We identified specific brain regions that were more closely related to the occurrence and development of SAP, and our probability map and box plot provided valuable insights into the distribution characteristics of ICH in patients with various types of pneumonia.

## Data availability statement

The original contributions presented in the study are included in the article/Supplementary material, further inquiries can be directed to the corresponding authors.

## Ethics statement

The studies involving human participants were reviewed and approved by Shengli Oilfield Central Hospital. The patients/participants provided their written informed consent to participate in this study. Written informed consent was obtained from the individual(s) for the publication of any potentially identifiable images or data included in this article.

## Author contributions

GY, MX, and XQ were responsible for the overall conception and supervision of the study. WC and XQ designed the study protocol. GY and MX conducted the experiments and drafted the initial manuscript. HS, YH, WC, and XQ contributed to critical revisions of the manuscript for important intellectual content. All authors have read and approved the final manuscript.
