# Supplementary material for: A brain CT-based approach for predicting and analyzing stroke-associated pneumonia from intracerebral hemorrhage
Source: Front Neurol. 2023 Jun 2;14:1139048. doi: 10.3389/fneur.2023.1139048 (PMC10272424; doi:10.3389/fneur.2023.1139048)
Supplement: Supplementary file 1 [file Data_Sheet_1.PDF]

Supplementary Material

1 SUPPLEMENTARY TABLES AND FIGURES

Table S1. The meanings of obtained features.

Name	Meaning	Name	Meaning
1	Proportion of ICH in Left-Cerebral-White-Matter	23	Proportion of ICH in Right-Inf-Lat-Ventricle
2	Proportion of ICH in Left-Cerebral-Cortex	24	Proportion of ICH in Right-Cerebellum-White-Matter
3	Proportion of ICH in Left-Lateral-Ventricle	25	Proportion of ICH in Right-Cerebellum-Cortex
4	Proportion of ICH in Left-Inf-Lat-Ventricle	26	Proportion of ICH in Right-Thalamus
5	Proportion of ICH in Left-Cerebellum-White-Matter	27	Proportion of ICH in Right-Caudate
6	Proportion of ICH in Left-Cerebellum-Cortex	28	Proportion of ICH in Right-Putamen
7	Proportion of ICH in Left-Thalamus	29	Proportion of ICH in Right-Pallidum
8	Proportion of ICH in Left-Caudate	30	Proportion of ICH in Right-Hippocampus
9	Proportion of ICH in Left-Putamen	31	Proportion of ICH in Right-Amygdala
10	Proportion of ICH in Left-Pallidum	32	Proportion of ICH in Right-Accumbens
11	Proportion of ICH in 3rd-Ventricle	33	Proportion of ICH in Right-Ventral-DC
12	Proportion of ICH in 4th-Ventricle	34	Proportion of ICH in Right-Vessel
13	Proportion of ICH in Brain-Stem	35	Proportion of ICH in Right-Choroid-Plexus
14	Proportion of ICH in Left-Hippocampus	F/B	The proportion of the volume of cerebrospinal fluid to the volume of brain parenchyma
15	Proportion of ICH in Left-Amygdala	L/B	The proportion of the volume of ICH to the volume of brain parenchyma
16	Proportion of ICH in Left-Accumbens	L/F	The proportion of the volume of ICH to the volume of cerebrospinal fluid
17	Proportion of ICH in Left-Ventral-DC	L/All	The proportion of the volume of ICH to the volume of brain
18	Proportion of ICH in Left-Vessel	F/All	The proportion of the volume of ICH to the volume of brain
19	Proportion of ICH in Left-Choroid-Plexus	fluid	Volume of the cerebrospinal fluid
20	Proportion of ICH in Right-Cerebral-White-Matter	brain	Volume of the brain parenchyma
21	Proportion of ICH in Right-Cerebral-Cortex	lesion	Volume of the ICH
22	Proportion of ICH in Right-Lateral-Ventricle		

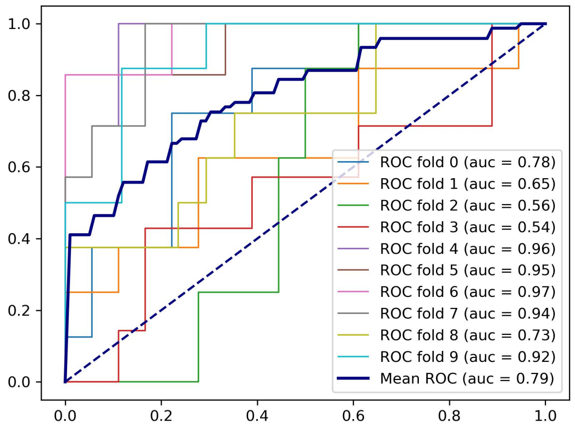

Figure 1a. This is Subfigure 1.

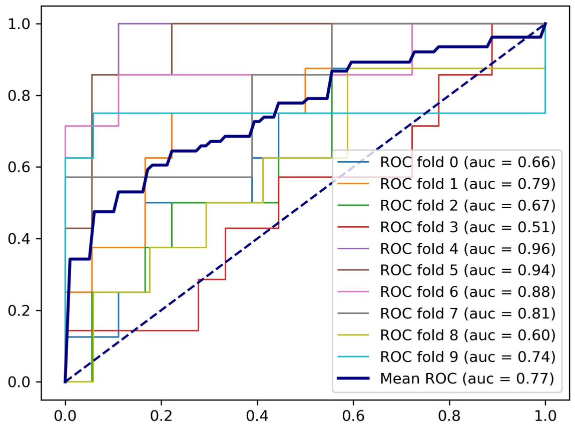

Figure 1b. This is Subfigure 2.

Figure 1. ROC curves of logistic regression model. **(A)** For predicting SAP. **(B)** For predicting SAP above moderate level.

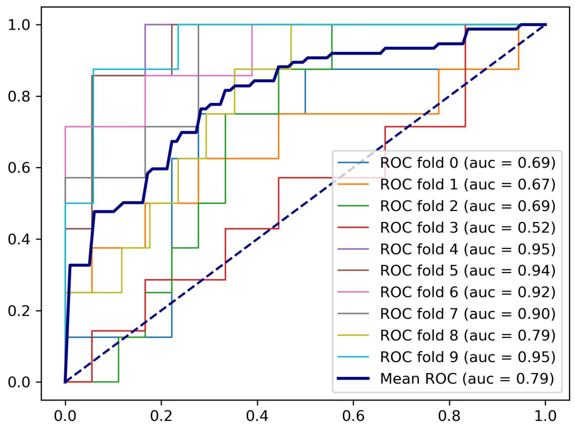

Figure 2a. This is Subfigure 1.

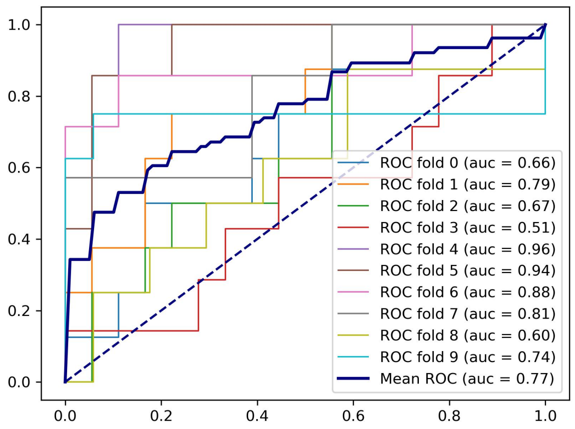

Figure 2b. This is Subfigure 2.

Figure 2. ROC curves of SVM model. **(A)** For predicting SAP. **(B)** For predicting SAP above moderate level.
